# Perceptions of Modulatory Factors in Migraine and Epilepsy: A Multicenter Study

**DOI:** 10.3389/fneur.2021.672860

**Published:** 2021-06-03

**Authors:** Emel Ur Özçelik, Katia Lin, Ruta Mameniškienè, Juiane Sauter Dalbem, Heloise Helena Siqueira, Rūta Samaitienė, Luz Eleonora Vega Zeissig, Armando Ferreira Fonseca, Juliana Mazini Alves, Mariana dos Santos Lunardi, Luiz Paulo de Queiroz, Erika Zubavičiūtė, Peter Wolf, Betül Baykan

**Affiliations:** ^1^Departments of Neurology and Clinical Neurophysiology, Istanbul Faculty of Medicine, Istanbul University, Istanbul, Turkey; ^2^Department of Neurology, Universidade Federal de Santa Catarina, Florianópolis, Brazil; ^3^Centre for Neurology, Vilnius University, Vilnius, Lithuania; ^4^Department of Neurology, Universidade Federal de Mato Grosso, Cuiabá, Brazil; ^5^Faculty of Medicine, Clinic of Children's Diseases, Vilnius University, Vilnius, Lithuania; ^6^Centro de Epilepsia Humana y Neurocirurgia Funcional, Guatemala City, Guatemala; ^7^Faculty of Medicine, Vilnius University, Vilnius, Lithuania; ^8^Danish Epilepsy Center, Dianalund, Denmark

**Keywords:** migraine, epilepsy, exogenous modulators, precipitant factors, inhibitory factors, self-awareness

## Abstract

**Background:** Migraine and epilepsy are both common episodic disorders, typically precipitated or inhibited by some modulatory factors (MFs).

**Objective:** To assess the self-perception of MFs in patients with migraine (PWM) compared to patients with epilepsy (PWE) with a standardized protocol in different countries.

**Methods:** Transcultural multicenter comparative cross-sectional study. All consecutive patients who fulfilled the ICHD-3 criteria for migraine and ILAE's criteria for epilepsy, with at least 1 year of follow-up were interviewed with a semi-structured questionnaire on clinical and epidemiological data and were asked to identify all experienced MFs from a provided list.

**Results:** A total of 608 individuals were surveyed at five university referral centers in Brazil, Guatemala, Lithuania and Turkey. Two hundred and nineteen (91.6%) PWM and 305 (82.7%) PWE identified attack precipitating factors (PFs; *p* < 0.001). The most frequent three PFs reported by epilepsy patients were: “lack of sleep” (56.6%), “emotional stress” (55.3%), “negative feelings” (53.9%), while among migraine patients “emotional stress” (81.6%), “lack of sleep” (77.8%), “negative feelings” (75.7%) were cited. Inhibitory factors (IFs) for the episodes were reported by 68 (28.5%) PWM and 116 (31.4%) PWE. “Darkness” was the most common one, described by 35.6% of PWM whereas “positive feelings” reported by 10.6% of PWE. Most MFs are concordant across the countries but some transcultural differences were noted.

**Conclusion:** The MFs of migraine and epilepsy attacks and their varying frequencies according to different countries were investigated with the same standardized questionnaire, for the first time. MFs were recognized very often in both migraine and epilepsy cohorts, but in distinct disease-specific prevalence, being more frequent in migraine. Recognition of self-perceived MFs may be helpful for the management of both illnesses.

## Introduction

Migraine and epilepsy are the two most common paroxysmal disorders of the central nervous system, with a prevalence of ~12% or more (1, 2), and ~1–2 % (3, 4), respectively. Different as they are, they have traits in common. One of them is the challenge to cope with the unpredictability of episodes. To know factors that may facilitate or provoke an attack, especially avoidable factors, would be a great help, and many patients attempt to identify them as well as factors having the opposite effect of preventing or inhibiting their paroxysmal events (5). Their views on such modulatory factors (MFs) have been the object of several investigations. In these, the two conditions were studied separately with different questionnaires, preventing a direct comparison, which would be interesting for various reasons. First, clear differences between the two heterogeneous conditions would be expected. Then, both could have certain traits in common like influences of stress or menstruation. Finally, there could be both unexpected differences and likenesses that could provide new insights into the experience of living with a paroxysmal disorder and suggest new hypotheses on the pathophysiology of the respective attacks.

We decided to conduct for the first time a comparative investigation of both disorders with the same instrument. As a second objective of this study, we compared the MFs between subgroups of epilepsy (focal vs. generalized) and migraine (with aura vs. without aura). The transcultural design allowed us, in addition, to identify possible national or regional traditions. Our working hypothesis was that it would be possible to identify different, condition-specific patterns and views of modulation of paroxysms. The focus of our study is on patients' experiences rather than the identification of objective such modulators, although their existence seems today well-established by prospective investigations of both conditions (6, 7).

## Methods

### Subjects and Questionnaire

A multicenter observational study was undertaken in four university referral centers for neurological disorders located in four different countries, where total of 608 patients was interviewed from February 2016 to December 2019. All consecutive patients, who met the existing valid diagnostic criteria at the beginning of the study, were re-evaluated according to the new diagnostic criteria. The patients who fulfilled either the International Classification of Headache Disorders 3rd Edition-ICHD-3 (2018) criteria for episodic migraine or International League Against Epilepsy-ILAE's (2017) criteria for focal or generalized epilepsy were included for further analyses (8, 9). All participants should have at least 1 year of follow-up to secure the correct diagnoses and experienced a minimum of two attacks.

For a comparative study in migraine and epilepsy, the same instrument needed to be used for both. As this was not available, we developed a new questionnaire in several expert discussion rounds and based on the existing questionnaires (Supplementary Table 1). It was physician-administered in a semi-structured face-to-face interview. Patients were asked to identify from this list all factors (MFs) that they perceived to be associated with the occurrence of seizures/migraine episodes.

Given that, in this neglected field, there is at present no generally accepted terminology, we defined “modulation” as including both “precipitating factors (PFs)” and “inhibitory factors (IFs).” Triggers pointed out to the specific and rare sensory or cognitive factors immediately precipitating reflex epileptic seizures, which are not specifically sought for this study.

Patients were excluded from this protocol if they were <15 years old, had any cognitive deficit that could prevent them from understanding the questionnaire, had evidence of progressive structural central nervous system lesions or progressive encephalopathy, had coexisting conditions (epilepsy and migraine), and had non-epileptic events, such as psychogenic seizures.

For subgroup comparisons of migraine patients, those with at least two attacks with aura were included in the migraine with aura (MWA) group, and the others were included in the migraine without aura (MWOA) group. Epilepsy patients were divided into two main groups as focal and generalized epilepsy according to their syndromic diagnoses by experts, based on their electroclinical features.

This study was carried out in accordance with the Code of Ethics of the World Medical Association (Declaration of Helsinki, 2014); and institutional review boards and ethics committees for each site approved the study protocol: Universidade Federal de Santa Catarina Ethics Committee, CEPSH/UFSC N. 1.226.636 (14/09/2015); Istanbul Medical Faculty Ethics Committee, no. 26/02/16/262–04; Investigation Committee of the Epilepsy and Functional Neurosurgery Center Humana CENFHU-06-2015; and Vilnius Regional Biomedical Research Ethics Committee no. 158200-15-797-309, 2015-09-07. All subjects signed an informed consent form and voluntarily agreed to participate.

### Statistical Analyses

Statistical analysis was performed using IBM SPSS® Statistics Grad Pack software Premium version 26.0. Descriptive statistics were used to describe study population characteristics. Quantitative variables were expressed as mean ± standard deviation (SD), and qualitative variables were expressed as frequency and percentage values. The Shapiro–Wilk normality test was used to test the normality of the distribution of quantitative data. The independent samples *t*-test was used to compare normally distributed continuous variables, whereas the Mann–Whitney *U* and Kruskal–Wallis *H*-tests were used for variables not normally distributed. Pearson chi-square and Fisher's exact tests were used to compare categorical variables and frequencies of occurrence. While *p* < 0.05 value was considered statistically significant for epilepsy and migraine comparisons, Bonferroni-corrected *p*-value (0.05/2; *p* < 0.025) was considered significant for epilepsy and migraine subgroup comparisons. When the results between the comparisons were statistically significant, the parameter in the chi-square boxes that created the significance was determined according to the adjusted values; a value of ≥2 was considered as significant. In addition, the significant results for the most discordant MFs between various countries evaluated for triggers cited by more than 15 individuals to avoid bias.

## Results

Among the total of 608 subjects, 369 (60.7%) had epilepsy and 239 (39.3%) had migraine. The mean age was 33.24 ± 12.92 years (range 15–83), and 396 individuals (65.1%) were women (Table 1). The age distribution, duration of disease, and frequency of episodes were comparable, whereas the epilepsy group had an earlier age at onset and a lower rate of females. The main diagnoses were as follows: MWOA (53.6%), MWA (43.8%), and chronic migraine (2.6%) among patients with migraine (PWM); and focal epilepsy (64.5%) and generalized epilepsy (35.5%) among patients with epilepsy (PWE).

**Table 1:** Clinical and demographic information.

**a)**		**Epilepsy (*****N*** **= 369)**		**Migraine (*****N*** **= 239)**		***p***
Female		200 (54.2%)		196 (82.0%)		<0.001^a^^*^
Age (mean ± SD)		33.9 ± 13.3		32.1 ± 12.2		0.127
Age of disease onset (mean ± SD)		15.1 ± 9.6		22.1 ± 10.5		<0.001
Duration of disease (mean ± SD)		17 ± 11.6		15.1 ± 13.6		<0.006
Monthly episode frequency (mean ± SD)		7.2 ± 24.1		8.6 ± 6.6		<0.001
**b)**		**Brazil (*****N*** **= 82)**	**Turkey (*****N*** **= 96)**	**Guatemala (*****N*** **= 42)**	**Lithuania (149)**	***p***
Epilepsy (*N* = 369)	Age (mean ± SD)	38.4 ± 11.9	33.6 ± 10.3	27.9 ± 9.4	31.2 ± 14.7	<0.001^c^
	Duration of disease (mean ± SD)	21.1 ± 12.8	14.2 ± 10.7	15.1 ± 9.8	12.6 ± 11.4	<0.001^c^
**c)**		**Brazil (*****N*** **= 44)**	**Turkey (*****N*** **= 94)**	**Guatemala (*****N*** **= 0)**	**Lithuania (*****N*** **= 101)**	***p***
Migraine (*N* = 239)	Age (mean ± SD)	39.9 ± 13.1	35.3 ± 10.5	-	25.8 ± 10.1	<0.001^c^
	Duration of disease (mean ± SD)	21.1 ± 15.5	11.2 ± 10.2	-	-	<0.001^b^

*SD, standard deviation*.

a *Pearson's chi-square*.

b *Mann–Whitney U-test*.

c *Kruskal–Wallis H-test*.

* *Statistically significant (p < 0.05)*.

**Figure 1:**
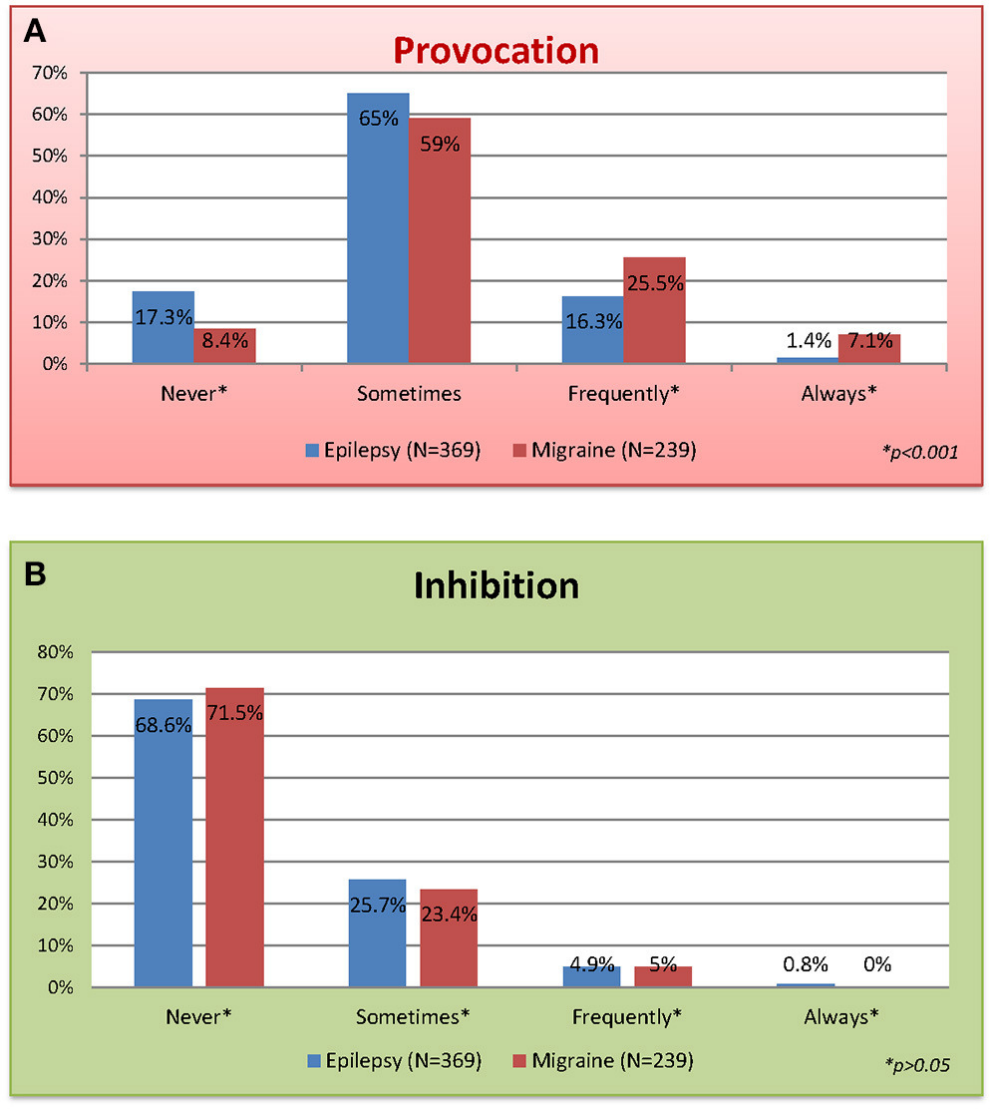
Lifetime self-reported modulation of seizures or migraine episodes: **(A)** provocation and **(B)** inhibition.

The occurrence of PFs appeared very common in both groups, but it was reported more often with migraine (91.6%) than with epilepsy (82.7%, *p* < 0.001, Figure 1A). Likewise, patients with both conditions experienced IFs in comparable but much lower frequency. IFs were not reported by 171 (71.5%) PWM and 253 (68.6%) PWE (Figure 1B). Additionally, the time lapse between the stimulus and the seizure or migraine episode is detailed in Table 2.

**Table 2:** Delay between the stimulus and the episode of seizure/migraine.

**Time**		**Epilepsy (*N* = 369) %**	**Migraine (*N* = 239) %**
	Unknown^*^	**18.4**	11.7
	Immediate	8.7	8.4
	Seconds	9.8	10.5
	Minutes	22.8	20.1
	Hours^*^	13.8	**33.9**
	Variable	19	15.1
	Not applicable	7.5	0.3

*The bold and underlined ones show statistically significant difference between the columns according to the adjusted residual (≥2) values in Pearson's chi-square*.

* *p < 0.001*.

**Figure 2:**
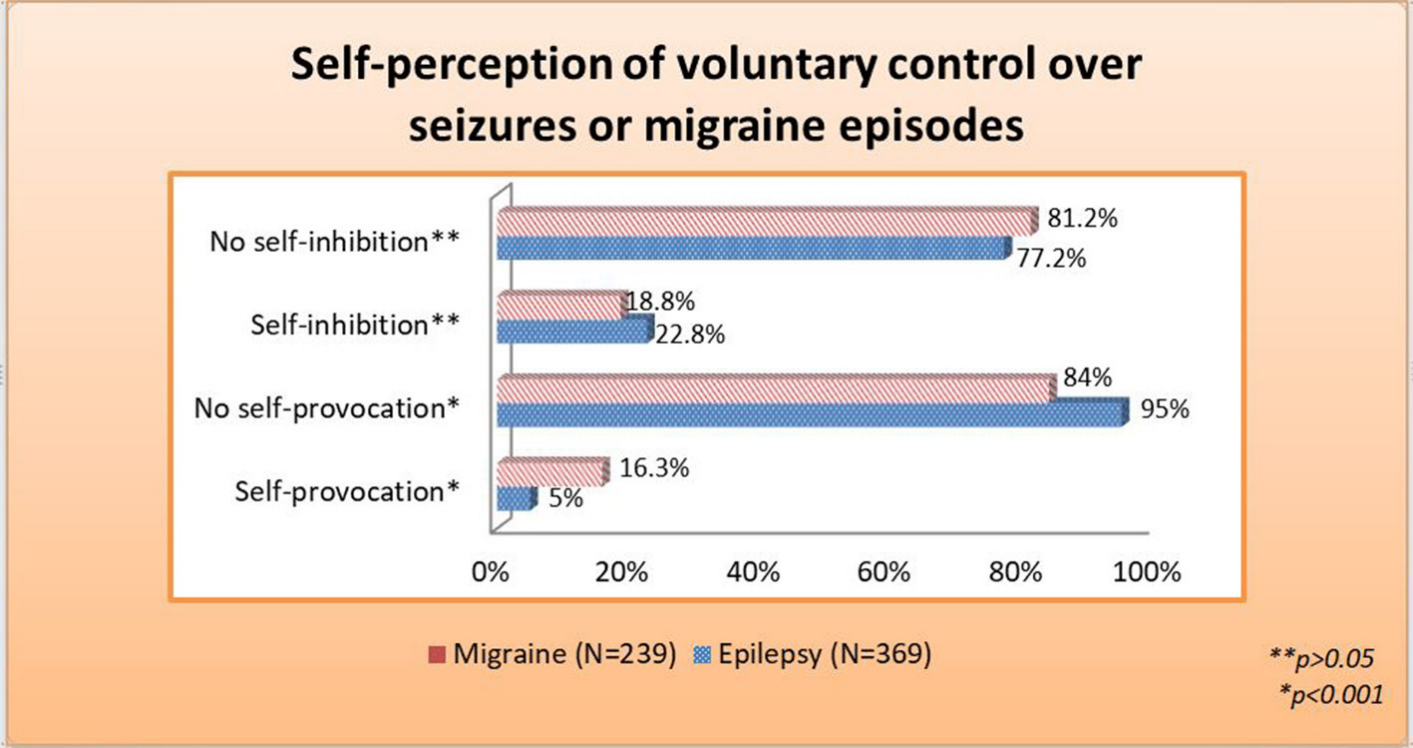
Self-perception of voluntary control over seizures or migraine episodes.

Moreover, substantial numbers of 82 (22.8%) PWE and 45 (18.8%) PWM declared to be able to prevent or arrest their episodes (Figure 2), whereas the counterpart, i.e., the ability to provoke attacks, was reported by 16.3% of PWM and, significantly less frequently, 5% of PWE.

There were statistically significant differences between the epilepsy and migraine groups regarding the various types of PFs (*p* < 0.05). Migraineurs were more sensitive to many of those triggers related to emotional and physical conditions, concentration-required activities, and also sleep, hormonal, and dietary changes. The comprehensive table detailing all PFs and IFs from the checklist can be found as Supplementary Table 2, and we will report here the leading items.

The five main PFs reported were as follows: lack of sleep (56.6%), emotional stress (55.3%), negative feelings (53.9%), physical stress (29.5%), and certain thoughts (20.3%) by epilepsy patients, while emotional stress (81.6%), lack of sleep (77.8%), negative feelings (75.7%), hunger (71.1%), and fasting (59%) by migraine patients. Several other PFs appeared rather common in both conditions but with different weight. Provocation of attacks with alcohol reported by 42.1% of PWM and 21.5% of PWE, flickering lights by 52.7% of PWM and 18.7% of PWE, television by 29.7% of PWM and 11.2% of PWE, and computer work by 43.5% of PWM and 13% of PWE. Excess sleep was reported by 54.8% of PWM but only 8.4% of PWE and fever by 30.1% of PWM and 13.5% of PWE. Menstruation was reported more frequently among migraineurs with active menstruation (68.4% of PWM vs. 18.9% of PWE).

**Figure 3:**
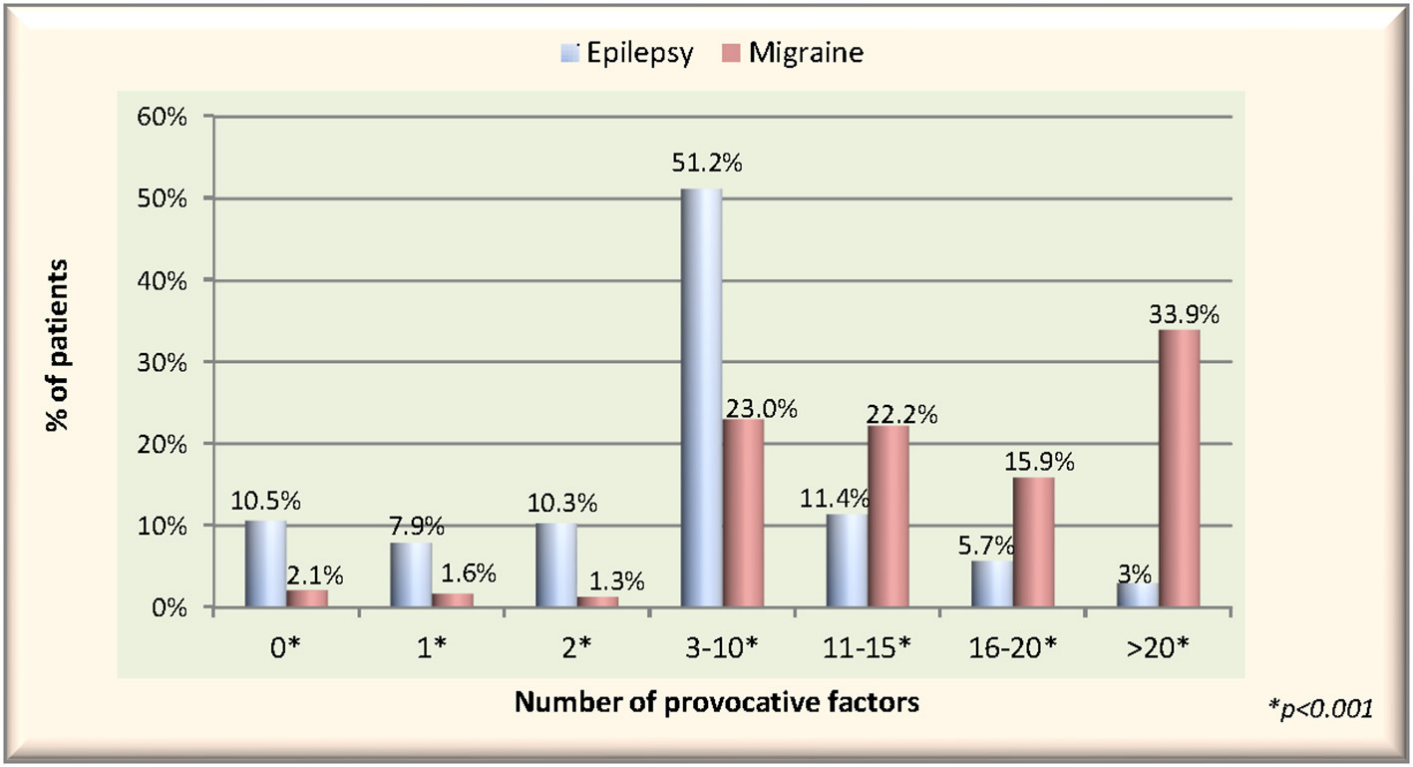
The rates of reported number of provocative factors per patient in migraine vs. epilepsy groups.

The rate of patients reporting at least two PFs was 81.6% in PWE, while it was 96.3% in PWM. Among migraineurs, the rate of those who reported at least 10 or more PFs was 72%, while in PWE, this rate was only 20.1%. There was a statistically significant difference between the number of reported factors per patient between two groups (*p* < 0.001) (Figure 3).

The main IFs reported by epilepsy patients were as follows: positive feelings (10.6%), thinking/concentration (7.3%), drawing (4.1%), sports (4.1%), and mental calculation (3.8%). The rates of IFs among migraine patients were as follows: darkness (35.6%), closing the eyes (31.4%), bathing/shower/hot water (18.4%), positive feelings (16.3%), and coffee (16.4%). The number of IFs reported per patient ranged from 1 to 17 in both groups, while the proportion of those reporting at least two IFs was 4.1% in PWE and 12.6% in PWM. The number of IFs reported per patient in migraine patients was significantly higher than in PWE (*p* < 0.001).

There was no significant difference between male and female genders, in terms of reporting provocation by triggers for their episodes in patients with both epilepsy and migraine (*p* = 0.298, *p* = 0.105, respectively). Also, there was no significant difference between women and men with epilepsy who reported that their attacks were inhibited by various factors (*p* = 0.561). However, the rate of men who reported that their migraine attacks were never inhibited by any factor (88.4%) was significantly higher than women (67.9%) (*p* = 0.005).

To compare the effect of age on triggers, PWE were divided into two groups: under 32 years old and above, according to the median value of the whole group. There was no statistically significant difference between the two age groups in terms of reporting provocation and inhibition; 21.3% of those above the age of 32 years and 11.6% of below the age of 32 stated that their attacks were frequently provoked by various factors (*p* = 0.083). Statistically significant ones are given in Table 3. Also, migraineurs were divided into two groups under the age of 30 and above, according to the median value of the whole group. Although there was no difference in terms of the responses given when asked about the rates of provoked and inhibited attacks (0.972), it was noteworthy that the young migraineurs were clearly more sensitive when all the MFs were questioned one by one. Statistically significant ones are given in Table 4.

**Table 3:** Modulatory factors with significant difference between two age groups in migraine.

	**PWM ≤ 30 years (*****N*** **= 122)**			**PWM > 30 years (*****N*** **= 117)**		
	**Provoc%**	**Inhibit%**	**NoMod%**	**Provoc%**	**Inhibit%**	**NoMod%**
Positive feelings^**^	3.3	**23.0**	**73.8**	8.5	9.4	82.1
Pleasant taste^**^	8.2	**9.8**	82	12.8	0.9	86.3
Chewing p = 0.000^*^	**17.2**	4.9	77.9	3.4	0.9	**95.7**
Unpleasant aroma^**^	30.2	–	**69.8**	**60**	–	40
Specific voices^**^	**29.5**	–	70.5	14.5	–	**85.5**
Certain rhythms^**^	15.6	**5.7**	78.7	18.8	0	81.2
Pain^*^	**44.3**	3.3	52.5	26.5	0	**73.5**
Public speaking^*^	**32.8**	0	67.2	12.8	0.9	**86.3**
Writing^*^	**69.6**	**6.6**	80.3	30.4	0	**94**
Drawing^**^	3.3	**9.8**	49.1	6	0	50.9
Singing^**^	8.2	**7.4**	84.4	5.1	0.9	90.6
Sexual activity^**^	6.8	**16.2**	76.9	12.3	3.5	84.2
Orgasm^**^	6	**16.2**	77.8	6	3.5	89.5
Excess sleep^**^	**63.9**	0.8	35.2	45.3	0	**54.7**
Seizures while asleep^**^	**37.7**	5.7	56.6	19.7	5.1	75.2
Fever^*^	**42.6**	0.8	56.6	17.1	0	**82.9**

*A comparison of the two groups here was made according to the median age of migraineurs. Statistical calculations were done among those who were exposed to modulatory factors. The bold and underlined ones are statistically significant rates according to the adjusted residual (≥2) values, and Bonferroni-corrected p-values were calculated according to number of migraine subgroups (0.05/2; p < 0.025) in Pearson chi-square*.

*Provoc, provocation; Inhibit, inhibition; NoMod, no modulation*.

* *p ≤ 0.001*.

** *p < 0.025*.

**Table 4:** Modulatory factors with significant difference between two age groups in epilepsy.

	**PWE ≤ 32 years (*****N*** **= 190)**^**≠**^			**PWE > 32 years (*****N*** **= 178)**^**≠**^		
	**Provoc%**	**Inhibit%**	**NoMod%**	**Provoc%**	**Inhibit%**	**NoMod%**
Certain memories^*^	12.1	2.6	85.3	**26.4**	0	73.6
Certain thoughts^**^	14.2	3.2	82.6	**26.4**	1.1	72.5
Negative feelings^**^	46.8	1.1	**52.1**	**61.2**	0	38.8
Lights^**^	**23.7**	0.5	75.8	13.5	0	86.5
TV^*^	**15.3**	1.1	83.7	6.2	0	93.8
Listening to talks, audit. overexposure^**^	3.7	1.6	94.7	**11.2**	0	88.8
Videogames, playst., game boy^**^	**16.4**	1.6	82	4.1	0	95
Working on computer^**^	**18.7**	3.3	78	8.7	1.2	90
Dancing^**^	2.2	**3.2**	94.6	0.6	0	99.4
Sexual activity^*^	1.7	**2.8**	95.5	**7.4**	0	92.6

*A comparison of the two groups here was made according to the median age of epilepsy patients. Statistical calculations were done among those who were exposed to modulatory factors. The bold and underlined ones are statistically significant rates according to adjusted residual (≥2) values, and Bonferroni-corrected p-values were calculated according to number of epilepsy subgroups (0.05/2; p < 0.025) in Pearson chi-square*.

*Provoc, provocation; Inhibit, inhibition; NoMod, no modulation; Audit, auditory; Playst, PlayStation*.

* *p ≤ 0.001*.

** *p < 0.025*.

≠ *Missing age data for one patient*.

**Figure 4:**
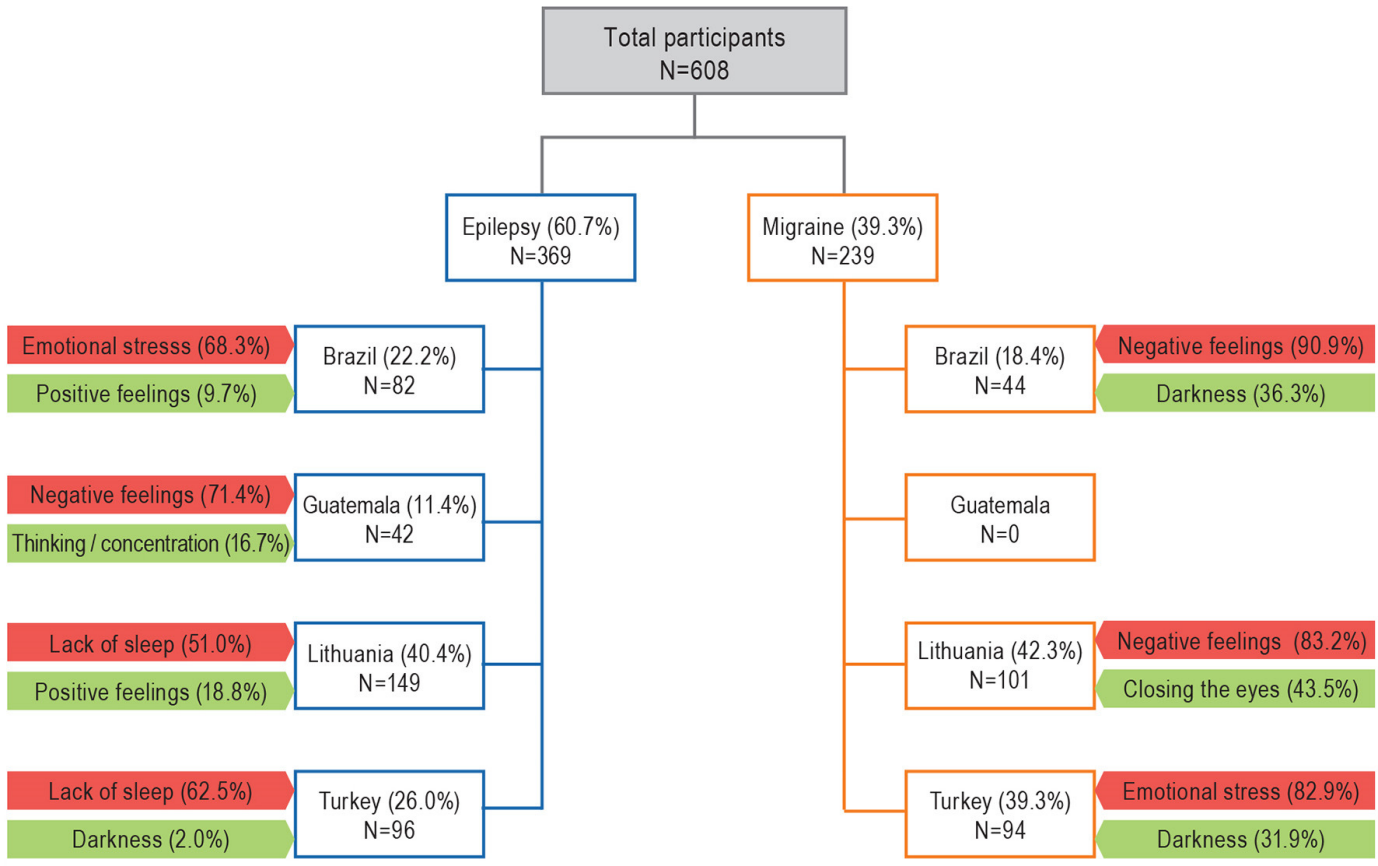
Interviewed patients and most frequently reported modulatory factors according to country. The red-painted boxes show the rate of those reporting “provocations,” whereas the green ones show the rate of reported “inhibitions”.

The top of the reported MFs according to country are given in Figure 4. While for PWE, emotional stress/negative feelings and lack of sleep were the most concordant PFs among countries, positive feelings were the most concordant common IF. Similarly, for PWM, emotional stress/negative feelings were the most concordant common PFs among countries, whereas darkness was the most concordant common IF.

In subgroup comparisons of epilepsy and migraine, there were also statistically significant differences for the various types of MFs. Lack of sleep and lights were the leading triggers reported among generalized epilepsies, whereas emotional and mnemonic ones were frequent among focal epilepsies (*p* < 0.025) (Table 5). Migraineurs with aura were clearly more sensitive to MFs than without aura (*p* < 0.025) (Table 6).

**Table 5:** Modulatory factors with significant difference between focal and generalized epilepsy.

	**Focal (*****N*** **= 238)**			**Generalized (*****N*** **= 131)**		
	**Provoc%**	**Inhibit %**	**NoMod %**	**Provoc%**	**Inhibit%**	**NoMod%**
Certain memories^*^	**24.4**	0.8	74.8	9.9	2.3	**87.8**
Certain thoughts^**^	**25.2**	1.3	73.5	11.5	3.8	**84.7**
Déjà vu^*^	**19.7**	0	80.3	7.6	1.5	**90.8**
Positive feelings^**^	**13**	10.1	76.9	2.3	11.5	86.3
Lights^*^	13	0.4	**86.6**	**29**	0	71
Flashes^*^	12.2	0.8	**87**	**30.5**	0	69.5
Brightness^**^	5.9	0.4	**93.7**	**14.5**	0	85.5
Listening to talks^**^	**9.7**	0	90.3	3.8	**2.3**	93.9
Sports^**^	**16**	3.8	80.3	3.8	4.6	**91.6**
Lack of sleep^**^	50.8	0.8	48.3	**67.2**	1.5	31.3
Seizure upon awakening^*^	12.6	0	**87.4**	**28.2**	1.5	70.2
Substance use^**^	4.5	0	95.5	3.9	**5.3**	90.8

*Statistical calculations were done among those who were exposed to modulatory factors. The bold and underlined ones are statistically significant rates according to adjusted residual (≥2) values, and Bonferroni-corrected p-values were calculated according to number of epilepsy subgroups (0.05/2; p < 0.025) in Pearson chi-square*.

*Provoc, provocation; Inhibit, inhibition; NoMod, no modulation*.

* *p ≤ 0.001*.

** *p < 0.025*.

**Table 6:** Modulatory factors with significant difference between migraine with and without aura.

	**MWOA**^**≠**^ **(*****N*** **= 133)**			**MWA**^**≠**^ **(*****N*** **= 104)**		
	**Provoc%**	**Inhibit%**	**NoMod%**	**Provoc%**	**Inhibit%**	**NoMod%**
Certain memories^*^	9	0	**91**	**21.2**	1	77.9
Chewing^*^	6.8	0.8	**92.5**	**15.4**	5.8	78.8
Unpleasant taste^*^	5.3	1.5	**93.2**	**15.4**	0	84.6
Lights^**^	41.4	0	**58.6**	**68.3**	1	30.8
Flashes^**^	30.1	0	**69.9**	**52.9**	1	46.2
Brightness^*^	30.1	0.8	**69.2**	**48.1**	1	51
Striped patterns^*^	14.3	0.8	**85**	**29.8**	0	70.2
Any song^**^	6.8	0	**93.2**	**31.7**	0	68.3
Certain rhythms^*^	12	1.5	**86.5**	**24**	4.8	71.2
Chess, cards, other^*^	4.2	0	**95.8**	**11.9**	**3.6**	84.5
Sexual activity^*^	6.9	6.2	**86.9**	13.1	**15.2**	71.7
Orgasm^*^	4.6	6.2	**89.2**	9.1	**15.2**	75.8
Physical stress^*^	50.4	0	**49.6**	**64.4**	1.9	33.7

*Statistical calculations were done among those who were exposed to modulatory factors. The bold and underlined ones are statistically significant rates according to adjusted residual (≥2) values, and Bonferroni-corrected p-values were calculated according to number of migraine subgroups (0.05/2; p < 0.025) in Pearson chi-square*.

*MWOA, migraine without aura; MWA, migraine with aura; Provoc, provocation; Inhibit, inhibition; NoMod, no modulation*.

≠ *Two uncertain diagnosis in the migraine group*.

* *p < 0.025*.

** *p ≤ 0.001*.

The significant results for the most discordant MFs between various countries, evaluated for triggers cited by more than 15 individuals to avoid bias, were given in Tables 7, 8.

**Table 7:** Discordant rates of attack modulatory factors in patients with epilepsy among the different countries.

	**Brazil *N* = 82**	**Turkey *N* = 96**	**Guatemala *N* = 42**	**Lithuania *N* = 149**
Certain memories^*^	**50.0**	3.1	19	12.8
Certain thoughts^*^	**53.7**	5.2	14.3	13.4
Déjà vu^*^	**24.4**	0	9.5	**22.1**
Positive feelings—inhibition^*^	9.8	0	0.8	**18.8**
Negative feelings^*^	**68.3**	53.1	**71.4**	41.6
Thinking/concentration ^*^	**34.1**	6.3	19	9.4
Inhibition^*^	3.7	0	*16.7*	**11.4**
Mental calculations^*^	**19.5**	1	7.1	4
Alcohol^*^	19.5	12.5	9.5	**27.5**
(Not applicable)	24.4	66.7	0	(2.7)
Coffee^**^	6.3	1	0^*^	**11.8**
(Not applicable)	3.7	4.2	0	(4)
Sudden unexpected loud noise^*^	**23.2**	0	14.3	*18.1*
Listening to talks, audit. overexposure ^*^	**22**	0	0	6.7
Pain^*^	**26.8**	0	*26.2*	15.4
Emotional speaking^**^	6.1	3.1	*16.7*	**15.3**
Reading silently^*^	7.3	0	4.8	**15.3**
Inhibition^*^	0	0	0	**11.9**
Sports^*^	**23.2**	1	14.3	11.4
Excess sleep^*^	**19.5**	0	0	10.1
Seizures upon awakening^**^	17.1	**27.1**	*31*	9.4
Seizures while asleep^*^	**22**	3.1	2.4	13.4
Physical stress^*^	**40.2**	**43.8**	9.5	20.1
Fever^**^	**22**	7.3	19	11.4

*Statistical calculations were done among those who were exposed to modulatory factors. The rates show provocation rates unless otherwise stated. The bold and underlined ones are statistically significant rates according to adjusted residual (≥2) values and p-value (<0.05) in Pearson chi-square. The italic and underlined ones are meaningful according to adjusted residuals and p-values (<0.05) in chi-square, but not the rates in which the number of individuals are <15*.

* *p ≤ 0.001*.

** *p < 0.05*.

**Table 8:** Discordant rates of attack modulatory factors in patients with migraine among the three different countries.

	**Brazil (*N* = 44) %**	**Turkey (*N* = 94) %**	**Lithuania (*N* = 101) %**
Certain memories^*^	*20.5*	5.3	**19.8**
Certain thoughts^*^	**40.9**	7.4	**44.4**
Positive feelings * inhibition	13.6	3.2	**29.7**
Negative feelings^*^	**90.9**	60.6	**83.2**
Thinking/concentration^*^	**61.4**	33	**62.4**
Decision-making^**^^≠^	**47.7**	27.7	25.7
Alcohol^*^	18.2	**14.9**	**44.6**
(Not applicable)	27.3	70.2	(2)
Coffee^*^	14.6	11.9	**21.8**
Inhibition	*26.8*	7.1	19.8
(Not applicable)	6.8	10.6	(0)
Smoking^*^	0	21.4	**33.3**
(Not applicable)	61.4	55.3	(2)
Another subst^*^	6.8	0	**15.3**
(Not applicable)	93.2	98.9	(3)
Chewing^*^	4.5	0	**22.8**
Fasting^**^	**81.8**	54.3	53.5
Pleasant taste^*^	2.3	**22.3**	3
Special taste or aroma^*^	**81.8**	27.7	54.5
Flashes^**^	36.4	28.7	**51.5**
Closing the eyes (Inhibition)^**^	27.3	20.2	**43.6**
TV^*^	38.6	10.6	**43.6**
Any song^*^	**40.9**	0	0
Specific pitch^*^	**40.9**	5.3	**44.6**
Listening to talks, audit. overexposure^*^	**65.9**	18.1	43.6
Specific voices^*^	11.4	3.2	**44.6**
Phone ringing, answering, etc.^*^	15.9	7.4	**34.7**
Certain rhythms^*^	**34.1**	2.1	23.8
Pain^*^	10.9	0.4	**24.3**
Certain own movements^*^	**45.5**	1.1	31.7
Videogames, playstation^*^	7	*21.6*	**26.7**
(Not applicable)	40.9	60.6	(1)
Work on computer^*^	35.8	37.3	**61.4**
(Not applicable)	11.4	20.2	(0)
Public speaking^*^	6.8	5.3	**46.5**
Writing^*^	*13.6*	2.1	**14.9**
Bathing, hot water inhibition^*^	9.1	6.4	**33.7**
Sports^*^	15.9	13.8	**25.7**
Inhibition^*^	9.1	0	**14.9**
Singing^*^	2.3	0	**15.1**
Dancing^*^	6.8	0	**15.1**
Sexual activity inhibition^*^	0	0	**23.2**
Orgasm inhibition^*^	4.5	2.1	**23.2**
Excess sleep^**^	45.5	45.7	**67.3**
Seizures upon awakening^*^	**72.7**	3.2	**61.4**
Seizures while asleep^*^	25	0	**57.4**
Physical stress^*^	**81.8**	34	65.3
Emotional stress^**^	**93.2**	83	75.2
Fever^*^	22.7	3.2	**58.4**
Menstruation^*^	**86.1**	58.2	**68**
(Not applicable)	18.2	36.2	(1)

*Statistical calculations were done among those who were exposed to modulatory factors. The rates show provocation rates unless otherwise stated. The bold and underlined ones are statistically significant rates according to adjusted residual (≥2) values and p-value (<0.05) in Pearson chi-square. The italic and underlined ones are meaningful according to adjusted residuals and p-values (<0.05) in chi-square, but not the rates in which the number of individuals are <15*.

*Audit, auditory*.

≠ *Decision-making between different possible things to do*.

* *p ≤ 0.001*.

** *p < 0.05*.

## Discussion

This large-sized and comprehensive transcultural survey draws attention to the relatively neglected topic of MFs, either provocative or inhibitory, which were comparatively investigated in 369 PWE and 239 PWM, in four culturally different countries using the same standardized method. Most subjects reported PFs (91.6% of PWM and 82.7% of PWE) at least once in their lifetime. These numbers agree with the literature, varying from 60 to 100% for migraine patients (10–14) and 47 to 97% in PWE (15–21). The occurrence of MFs is well-known in both epilepsy and migraine but has not been comparatively assessed before. Migraine and epilepsy are chronic disorders with episodic attacks, and long recognized associations between them include some clinical features, including external and internal triggers, as well as some gene mutations (22, 23).

There is a well-known gender predominance in migraine worldwide, with women representing more than 80% of the patient population, similar to our study. In fact, migraine occurs approximately three times more often in women than in men (18, 24, 25). Sex hormonal changes have major impacts particularly on migraine during lifetime; but the underlying mechanisms are not illuminated yet (4, 26). Diversely, Christensen et al. evaluated gender differences in epilepsy, finding no overall difference, but in subsequent analyses, they found that genetic generalized epilepsies were more frequent in women than in men (27). Similar to our findings, Ferlisi and Shorvon did not observe differences in frequency or type of seizure precipitants with regard to gender (15).

Several authors reported stress as the most prevalent PF reported by PWE (16–21), while in our study, it was the most prevalent PF reported in migraine. The first ranking PF among PWE was lack of sleep, similar to da Silva Sousa et al. (20). Sleep deprivation was also cited by other authors (18, 19). Certain thoughts and certain memories were not commonly assessed in other publications. da Silva Sousa et al. reported specific thoughts/concentration being recognized by 23% of the patients (20). In our study, certain thoughts and memories were reported in 20.3 and 19.2%, respectively, of PWE.

Emotional stress was the most important PF reported by migraine patients (81.6%), like other studies, where stress was reported by 48–84% probably related to the somatic effect of hyperexcitability on the autonomic nervous system (9–12, 28, 29).

Our PWM also mentioned negative feelings as a PF in 75.7%. Hauge et al. found it in up to 58% (11). Also, lack of sleep was similarly reported high among 77.8% of migraineurs in our study; it was also identified in other surveys by 50–64% (28). Besides, the influence of dietary factors or fasting was cited by 44–58% (28). Within our population of PWM, 71.1% reported hunger and 59% fasting as a triggering factor. It was remarkable that the dietary control of individual-specific food triggers reduced the number of monthly attacks of migraine in a double-blind, crossover, randomized controlled trial, showing evidence for the importance of searching and controlling the relevant MF, as an additional management option (30).

As expected, modulation of attacks by light sources was identified in higher rates among PWM (52.7% of PWM vs. 18.7% of PWE), related possibly to photophobia in relation to migraine. Also, menstruation was a very important PF among PWM (68.4%) and of less importance among PWE (18.9%), as consistent with literature (26, 31). An interesting and unexpected significant difference between migraine and epilepsy triggers was “fever” (13.5% of PWE vs. 30.1% of PWM reported attacks triggered by fever). Fever is a well-known trigger in epilepsy (32), and also headaches related to fever were reported in the Headache Classification-ICHD (2018), but there is not much known about fever triggering migraine episodes (8).

In our subgroup comparisons of PWE, lights, lack of sleep, and seizure upon awakening were reported more frequently among generalized epilepsies, whereas emotional, movement, and auditory-related triggers were frequent among focal epilepsies, as reported by others in different studies (5, 33).

Our migraineurs with aura reported a more sensitive picture to triggers. More frequently reported PFs among migraineurs with aura were as follows: visual triggers like lights and striped patterns; physical stress; certain memories; some auditory triggers; and games like chess/cards. Sexual activity and orgasm reported to be both inhibitory and provocatory on attacks more frequently in MWA than MWOA. Similar to ours, Kelman reported also migraineurs with aura were more sensitive to some triggers including lights (12). But there are some other studies showing different results. Rasmussen and Olesen found the frequency of various PFs was higher in migraineurs without aura (34), and Russel et al. only found lights more frequent among migraineurs with aura (35).

Perhaps the most remarkable finding in this direct comparison of epilepsy and migraine triggers is the identical appearance of the three leading provocative factors: negative feelings, emotional stress, and lack of sleep. It can be discussed to what extent these are real factors or, rather, subjective attributions. That negative feelings and their counterpart, positive feelings (which have a similar place among inhibitors), that appear in these roles may appear trivial; they may very well be prodromal symptoms rather than modulating factors. But they may also reflect still unrecognized systemic dynamics as factors of symptom generation that are common to both disorders.

To discover factors specific for the different conditions, it may be that we have primarily to look into less frequent observations where migraine and epilepsy differ. Thus, when we disregard the commonly reported influences of positive and negative feelings and the apparently specific effect of coffee in migraine, it appears that IFs in epilepsy primarily have something to do with increased concentration and arousal, but those in migraine have something to do with relaxation and reduced sensory input.

Indeed, we were expecting to show that reported PFs would be less frequent and IFs would be more frequent among elderly PWE; however, we could not show this. Mnemonic triggers (26.4%), negative feelings (61.2%), auditory overexposure (11.2%), and sexual activity (7.2%) were more frequent PFs among the elderly PWE, whereas lights (23.7%), TV(15.3%), videogames (16.4%), and computer (18.7%) were reported PFs among the younger group. This photic sensitivity is a well-known phenomenon among the younger PWE, especially in the genetic generalized epilepsy (GGE), and our survey confirms this (36). Interestingly, dancing (3.2%) and sexual activity (2.8%) were IFs reported more frequently among the younger PWE group, although the rates were not high. It is obvious that systematic prospective studies are needed to investigate the frequency and characteristics of triggers in epilepsy at different age ranges and during the course of the disease.

Younger migraineurs were more sensitive to MFs. On the other hand, reported rates of IFs were not higher among elderly PWM, which we were expecting to be. For instance, emotional stress has not been reported at divergent rates between elderly and younger PWE groups, and what even more surprising was that positive feelings were reported more frequently as an IF among the younger PWM (23%). The other frequent IFs among younger PWM were as follows: sexual activity (16.2%), pleasant taste (9.8%), drawing (9.8%), singing (7.4%), certain rhythms (5.7%), writing (6.6%), and pain (3.3%). On the other hand, frequently reported PFs among younger PWE were writing (69.6 %), excess sleep (63.9%), pain (44.3%), public speaking (32.8%), seizures while asleep (37.7%), fever (42.6%), specific voices (29.5%), and chewing (17.2%). The only more frequently reported trigger among the elderly group was unpleasant aroma (60%). Most probably, these divergent rates among the younger and elderly groups may be associated with development of trigger avoidance or desensitization strategies by time. There is a lack of studies investigating the impact of age and disease duration on migraine triggers (37).

The incidence of MFs for migraine and seizures across different countries demonstrated the clinical consistency of these conditions. However, the top first PF and IF were slightly different across the sites, suggesting that regional cultural characteristics and beliefs may have influenced the answers (38, 39), as previously found by Asadi-Pooya and Sperling when they compared seizure precipitants between a Middle Eastern country (Iran) and a Western country (USA) (40). Interestingly, a series of remarkable differences were found between the four participating countries (see Tables 7, 8). Certain memories (50%) and thoughts (53.7%), mental calculations (19.5%), thinking/concentration (34.1%), fever (22%), pain (26.8%), sports (23.2%), excess sleep (19.5%), and auditory factors (23%) were reported strikingly higher as being provocative for seizures in Brazilian PWE than PWE in other countries for unknown reasons. In Lithuanian PWE, alcohol (26%), coffee (11.4%), emotional speaking (15.7%), and reading silently (15.3%) were conspicuous PFs, whereas in Turkish PWE, physical stress (40.2%) and awakening (27.1%) were the significantly noted features. Negative feelings was the most reported PF among Guatemalans (71.4%) and followed by the Brazilian (68.3%) PWE. The other interesting point was the higher reporting rates of positive feelings (18.8%), reading silently (11.9%), and thinking/concentration (11.4%) as IFs among the Lithuanian PWE.

Furthermore, when the reported MFs of PWM among countries were compared, there were also interesting differences. The triggering stimuli, like certain memories (19.8%), coffee (21.8%), another substance (14.9%), and chewing (22.8%) were significantly common among Lithuanian PWM; decision making (47.7%), fasting (81.8%), special taste or aroma (81.8%), any song (40.9%), specific pitch (40.9%), listening to talks (65.9%), certain rhythms (34.1%), certain own movements (45.5%), awakening (72.7%), and physical stress (81.8%) were reported in discordant rates among Brazilian PWM. In Turkish PWM, pleasant tastes (22.3%) were reported as provocatory in higher rates. It was also shown that the same factors can act in opposite directions; positive feelings (29.7%), coffee (19.8%), sports (14.9%), sexual activity (22.8%), and orgasm (22.8%) were the outstanding IFs reported to be higher among Lithuanian PWM. All these discordant rates might be explained by the sociocultural differences, the way of perceiving questions in four different languages, as the terms may have slightly different connotations. Additionally for those PWE, discordances might also be explained by the fact of different prevalence of focal and generalized epilepsies among patients from different countries [e.g., focal epilepsy was present in different rates among Brazilian (92.7%), Lithuanians (67.1%) Turkish (36.5%), and Guatemalans (64.3%)]. We also want to emphasize that our findings underlined the fact that individualized approach is important to help the patients.

A very important question is whether perceptions of protective factors enable patients to perceive some voluntary control over their seizures or migraine attacks (41, 42). Lunardi et al. found that 50.7% of the patients identified at least one IF in a cohort of temporal lobe epilepsy (17). Moreover, 82 (22.8%) PWE and 45 (18.8%) PWM reported being able to prevent or arrest their episodes, and results were concordant with a previous study, where 23% of the patients revealed they were capable of avoiding the occurrence of their seizures (20). Pinikahana and Dono revealed that 69.8% from an epilepsy research database indicated that they had tried at least one technique to stop a seizure, with resting, acute medication use, and relaxation being the most common (18). Another study reported that 47% of the subjects could sometimes stop their seizures from happening, mostly by relaxation techniques (21). The most commonly reported behaviors of stopping the migraine attacks are rather non-specific like isolation from light, sound (75–95%), lying down (65–89%), and sleeping (60–89.3%); and therefore, causality establishment is not easy. Thus, the exact rate of PWM who could stop their attacks could not be stated appropriately (42).

On the contrary, when the induction of seizures was evaluated, only 18 (5%) of PWE and 38 (16.3%) of those with migraine informed that they could provoke a seizure or migraine attack. Cull et al. reported that 8.9–9.7% of their cohort could induce seizures (43). Pinikahana and Dono revealed a larger number: about 35.1% of their population could trigger a seizure voluntarily (18). Self-provocation in migraine is a neglected issue, but it has been reported that attacks/auras could be triggered by pharmacological and non-pharmacological stimuli (44, 45). Hougaard et al. reported that only 11% of the attacks were triggered by exercise in the laboratory environment, but none by light stimulation in PWM, who already reported that their attacks were triggered by light and exercise in daily life (46). These studies reflect the complex nature of the initiation of migraine.

Our study has some limitations: First of all, to make the distinctions clearer, the highly prevalent situation of comorbidity between migraine and epilepsy was not systematically assessed in our population, and those cases with comorbidity were not included (47).The questionnaire used here was constructed as an instrument that could be applied for both investigated disease conditions, for the sake of this study. In addition, there is a lack of gold-standard tests to validate our questionnaire with. Although validation is recommended in all questionnaires in the health field to ensure that the questionnaire is psychometrically sound, it may be argued by some authors (48), and most of the questionnaires previously published on the subject of MFs have not been validated yet.

Theoretically, episode prediction currently rests on the identification of trigger factors and protective factors. This task may be misleading, as the terms “trigger factors” (“measureable endogenous or exogenous events/exposures associated with an increased probability of an attack over a relatively brief period of time, with examples such as menses”) and “premonitory features” (“precede the attack by up to 48 h and may include cognitive and behavioral factors, such as feeling tired/weary, concentration difficulties”) are often confused in the literature (39), compromising the reproducibility of these numbers across different studies.

Several items of our questionnaire could be criticized, though reflecting real-life usage, being not to clearly distinguish between these two possibilities. They were, however, kept in, as we otherwise could have lost important information on patients' subjective experiences. For instance, it is not clear if all patients from different countries with different diagnoses mean the same thing when they give an answer to a question, such as *déjà vu* or negative feelings. However, this is a limitation inherent to all questionnaires; they are all subject to patients' interpretation.

Furthermore, other limitations, characteristic of all psychosocial studies, are worthwhile to mention. As already said above, in studies like this based on self-reported or researcher-administrated surveys, the collected information may be influenced by beliefs, sociocultural levels, affective disorders, or symptoms and also recall bias. While questioning the triggers of the patients, it might be useful to correlate them with anxiety and depression scales, but as it was not our main purpose in this study, we did not accede to them. On the other hand, such as the predominance of women among the migraineurs, age of onset, and hence the disease duration, frequency of episodes was indeed different between groups since epilepsy and migraine have a little bit distinct nature. Also, in such a large series of cases, the non-matching age distribution and durations of diseases between countries can be considered among the limitations. Nonetheless, such studies are important for hypothesis generation, while being able to assess beliefs about triggers and premonitory features and multiple other factors. Besides, to identify MFs from a checklist may limit only to candidate factors with available data, although our questionnaire was intended to be comprehensive with more than 60 items. Also, this modality of study may not distinguish between causality and reverse causality (39), while it is not time-consuming and it may be easily applied in different and large populations. At last, this study was conducted mostly in tertiary referral centers, which may have biased the population toward more severe epilepsy or migraine conditions.

The reliable recognition of opportunities to reduce episode frequency such as trigger identification and avoidance, preemptive therapy (either pharmacologic or behavioral approaches), and enhancing timely protective factors may, in some cases, be the mainstay of the treatment, or being important as an adjunctive measure alongside the regular pharmacological treatment, since the unpredictability of migraine or seizure episodes is one of the most incapacitating features of these conditions (39, 49). Further studies assessing the roles of MFs in specific epilepsy syndromes as well as other migraine subtypes with more homogeneous patient populations will provide more useful information.

## Conclusion

The great majority of patients identified PFs for both their epileptic seizures and migraine attacks, while one in three patients recognized that their episodes could be inhibited by specific measures. PWM report higher sensitivity to triggers than the PWE, when investigated with the same standardized questionnaire, for the first time. Interestingly, the same stimulus can both precipitate and abort a seizure in different individuals or even in the same individual. This phenomenon does not have any explanation so far; it may depend on the state of cortical network activation at the moment the input is given in a susceptible person. A better understanding of these MFs may provide insights into disease pathophysiology, as also the knowledge of seizure precipitants may empower patients in increasing their self-awareness, by promoting behavioral modification with avoidance of specific high-risk situations and, potentially, a reduction in seizure/migraine episodes. We also showed that these MFs are mostly concordant across the countries only with some minor transcultural changes, which further indicated that they are reliable means for future investigations. Proper recognition of seizure/migraine trigger factors and other MFs could be helpful in routine daily practice, leading to new and personalized treatment strategies, besides helping to create homogenous groups for advanced genetic and neuroimaging researches.

## Data Availability Statement

The original contributions presented in the study are included in the article/Supplementary Material, further inquiries can be directed to the corresponding author/s.

## Ethics Statement

The studies involving human participants were reviewed and approved by Universidade Federal de Santa Catarina Ethics Committee, CEPSH/UFSC N. 1.226.636 (14/09/2015); Istanbul Medical Faculty Ethics Committee, n° 26/02/16/262–04; Investigation Committee of the Epilepsy and Functional Neurosurgery Center Humana CENFHU-06-2015; Vilnius Regional Biomedical Research Ethics Committee n° 158200-15-797-309, 2015-09-07. Written informed consent to participate in this study was provided by the participants' legal guardian/next of kin.

## Author Contributions

BB, EU, KL, RM, RS, and PW contributed to the conception and design of the study. EU, KL JS, HS, RM, RS, LV, AF, JA, MS, LQ, and EZ contributed to data collection. KL and EU organized the database and performed the statistical analysis. KL, EU, BB, and PW wrote the first draft of the manuscript. All authors contributed to manuscript draft, read, and approved the submitted version.

## Conflict of Interest

The authors declare that the research was conducted in the absence of any commercial or financial relationships that could be construed as a potential conflict of interest.

## Abbreviations

MFs: modulatory factors
IFs: inhibitory factors
PFs: precipitating factors
PWM: patients with migraine
PWE: patients with epilepsy
MWA: migraine with aura
MWOA: migraine without aura.
