# Supplementary material for: Perceptions of Modulatory Factors in Migraine and Epilepsy: A Multicenter Study
Source: Front Neurol. 2021 Jun 3;12:672860. doi: 10.3389/fneur.2021.672860 (PMC8209378; doi:10.3389/fneur.2021.672860)
Supplement: Supplementary file 1 [file Data_Sheet_1.docx]

**Supplementary**

**Box 1. Precipitant and inhibitory factors on epilepsy and migraine**

| ***Memories and feelings*** | ***Auditory and related stimuli*** | ***Physical conditions*** |
| --- | --- | --- |
| 1. Certainmemories | 31. Sudden unexpected loud noises | 62. Lack of sleep |
| 2. Certain thoughts | 32. Music: any | 63. Seizures/episodes at awakening |
| 3. Feeling of familiarity (*déjà vu*) | 33. Musical tones of a specific pitch | 64. Seizures/episodes at falling asleep |
| 4. Positive feelings (joy, pleasure, etc.) | 34. Listening to talks, auditory overexposure | 65. Physical stress  66. Emotional stress |
| 5. Negative feelings (fear, anxiety, depression, anger, etc.) | 35. Specific voices  36. Telephone (ringing, answering)  37. Playing a musical instrument  38. Certain rhythms | 67. Fever  68. Menstruation  69. Hunger  70. Other: open-end for writing |
| ***Mental activities*** |  | ________ |
| 6. Thinking, concentration | ***Other perceptions*** |  |
| 7. Mental calculation  8. Written calculation, calculation with abacus, computer | 39. Touch at specific places (by others or oneself)  40.Pain |  |
| 9. Decision-making between different possible things to do | 41. Certain own movements |  |
| 10. Decision-making between different approaches to a task | ***Games***  42. Chess, cards, others |  |
| ***Nutrition and related*** | 43. Videogames, Playstation, Gameboy, etc. |  |
| 11. Alcohol |  |  |
| 12. Coffee | ***Activities*** |  |
| 13. Smoking  14. Other recreative substances  15. Chewing  16. Swallowing | 44. Working on your computer (workor games)  45. Complex movements (handicrafts, etc.) |  |
| 17. Pleasant taste  18. Unpleasant taste  19. Neutral taste  20. Pleasant smell and aromas  21. Unpleasant smell and aroma  22. Neutral smell and aroma  ***Visual stimuli***  23. Flickering lights  24. Single flash  25. Passing from dark to light  26. Darkness  27. Closing the eyes  28. Striped patterns  29. Television  30. Specific images, faces, objects, forms | 46. Public speaking  47. Private speaking  48. Emotional speaking  49. Reading aloud  50. Reading silently  51. Writing  52. Drawing  53. Toothbrushing  54. Bathing, shower, hot water  55. Sports  56. Sing  57. Dance  58. Sexual activity  59. Orgasm  60. Micturition  61. Evacuation |  |

**Supplementary**

**Box 2. Modulatory factors with significant difference between epilepsy and migraine**

|  | **Epilepsy**  **N=369** | | | | **Migraine**  **N=239** | | | | | |  |
| --- | --- | --- | --- | --- | --- | --- | --- | --- | --- | --- | --- |
|  | **Provoc%** | **Inhib%** | **NoMod%** | | **Provoc%** | | **Inhib%** | | **NoMod%** | |  |
| **Certain thoughts^≠^** | 20.3 | 2.2 | **77.5** | | **29.1** | | 1.3 | | 69.6 | |  |
| **Feeling of familiarity^≠^** | **15.5** | 0.5 | 84.0 | | 4.2 | | 0.4 | | **95.4** | |  |
| **Negative feelings^≠^** | 53.9 | 0.5 | **45.6** | | **75.7** | | 0 | | 24.3 | |  |
| **Thinking/concentration^≠^** | 15.2 | **7.3** | **77.5** | | **50.6** | | 2.1 | | 47.3 | |  |
| **Mental calculation^≠^** | 7.0 | 3.8 | **89.2** | | **20.1** | | 1.7 | | 78.2 | |  |
| **Written calculations^≠^** | 6.5 | 2.7 | **90.8** | | **20.9** | | 0.4 | | 78.7 | |  |
| **Decision ..to do.^≠^** | 7.6 | 3.0 | **89.4** | | **30.5** | | 1.7 | | 67.8 | |  |
| **Decision ..approaches^≠^** | 7 | 3.5 | **89.5** | | **29.7** | | 1.3 | | 69 | |  |
| **Alcohol^,≠^** | 21.4 | 1.1 | **77.5** | | **42.1** | | 3.1 | | 54.8 | |  |
| **Coffee^≠^** | 6.4 | 0.6 | **93** | | **16.8** | | **16.4** | | 66.8 | |  |
| **Smoking^≠^** | 5.1 | 0.9 | **94** | | **26.6** | | **6.3** | | 67.1 | |  |
| **Another substance^≠^** | 4.3 | 1.5 | **94.2** | | **17.6** | | **4.9** | | 77.5 | |  |
| **Chewing^≠^** | 3 | 1.1 | **95.9** | | **10.5** | | 2.9 | | 86.6 | |  |
| **Swallowing^≠≠^** | 1.1 | 0.8 | **98.1** | | **4.2** | | 0.8 | | 95 | |  |
| **Fasting^≠^** | 6.1 | 0.8 | **93.1** | | **59.0** | | 0.4 | | 40.6 | |  |
| **Pleasant taste^≠^** | 1.3 | 1.1 | **97.6** | | **10.5** | | **5.4** | | 84.1 | |  |
| **Unpleasant taste^≠^** | 2.2 | 0.5 | **97.3** | | **9.6** | | 0.8 | | 89.5 | |  |
| **Pleasant aroma^≠^** | 1.5 | 1.5 | **97** | | **29** | | 0 | | 71 | |  |
| **Unpleasant aroma^≠^** | 6 | 0.9 | **93.1** | | **50.7** | | 0 | | 49.3 | |  |
| **Special taste or aroma^≠^** | 3.8 | 0.3 | **95.9** | | **49.0** | | 0.4 | | 50.6 | |  |
| **Lights^≠^** | 18.7 | 0.3 | **81** | | **52.7** | | 0.4 | | 46.9 | |  |
| **Flashes^≠^** | 18.7 | 0.5 | **80.8** | | **39.8** | | 0.4 | | 59.8 | |  |
| **Brightness^≠^** | 8.9 | 0.3 | **90.8** | | **37.7** | | 0.8 | | 61.5 | |  |
| **Darkness^≠^** | 3.2 | 1.4 | **95.4** | | 3.3 | | **35.6** | | 61.1 | |  |
| **Closing the eyes^≠^** | 1.9 | 3 | **95.1** | | 2.9 | | **31.4** | | 65.7 | |  |
| **Stripped patterns^≠^** | 5.7 | 0.5 | **93.8** | | **20.9** | | 0.4 | | 78.7 | |  |
| **TV^≠^** | 11.1 | 0.5 | **88.3** | | **29.7** | | 0.8 | | 69.5 | |  |
| **Sudden unexpected loud noises^≠^** | 14.1 | 0.3 | **85.6** | | **47.7** | | 0.8 | | 51.5 | |  |
| **Any song^≠^** | 5.1 | **2.5** | **92.4** | | **15.8** | | 0 | | 84.2 | |  |
| **Musical tones of a spesific pitch^≠^** | 4.9 | 1.3 | **93.8** | | **28.5** | | 1.7 | | 69.8 | |  |
| **Listening to talks, auditory overexposure^≠^** | 7.6 | 0.8 | **91.6** | | **37.7** | | 0.4 | | 61.9 | |  |
| **Specific voices^≠^** | 4.6 | 0.5 | **94.9** | | **22.2** | | 0 | | 77.8 | |  |
| **Telephone ringing-answering^≠^** | 2.4 | 0 | **97.6** | | **20.5** | | 0 | | 79.5 | |  |
| **Playing a musical instrument^≠^** | 1.7 | 2 | **96.3** | | **13.2** | | 1.3 | | 85.5 | |  |
| **Certain rhythms^≠^** | 2.2 | 1.4 | **96.5** | | **17.2** | | 2.9 | | 79.9 | |  |
| **Thouch at spesific places** | 3 | 2.2 | **94.8** | | **7.1** | | 5.9 | | 87 | |  |
| **Pain^≠≠^** | 15.2 | 1.4 | **83.5** | | **35.6** | | 1.7 | | 62.8 | |  |
| **Certain own movements^≠^** | 6 | 1.9 | **92.1** | | **22.2** | | 1.3 | | 76.6 | |  |
| **Videogames, playstation, Gameboy^≠≠^** | 11 | 0.9 | **88.1** | | **22.7** | | 0.6 | | 76.7 | |  |
| **Working or games on computer^≠^** | 14 | 2.3 | **83.7** |  | | **48.4** | | 0 | | 51.6 | |
| **Complex movements^≠^** | 2.7 | 2.4 | **94.9** |  | | **15.1** | | 7.9 | | 77 | |
| **Public speaking^≠^** | 8.1 | 1.4 | **90.5** |  | | **23** | | 0.4 | | 76.6 | |
| **Private speaking^≠^** | 3.2 | 2.2 | **94.6** |  | | **13.8** | | 2.5 | | 83.7 | |
| **Emotional speaking^≠^** | 8.6 | 0.7 | **90.7** |  | | **28.9** | | 0.4 | | 70.7 | |
| **Writing^≠^** | 2.2 | 1.4 | **96.5** |  | | **9.6** | | 3.3 | | 87 | |
| **Drawing^≠≠^** | 1.3 | 4.1 | **94.6** |  | | **4.6** | | 5 | | 90.4 | |
| **Reading aloud^≠^** | 4 | 1.4 | **94.6** |  | | **13** | | 1.2 | | 85.8 | |
| **Bathing, hot water^≠^** | 6.8 | 1.3 | **91.9** |  | | 5.4 | | **18.4** | | 76.2 | |
| **Sports^≠≠^** | 11.7 | 4.1 | **84.2** |  | | **19.2** | | 8 | | 72.8 | |
| **Singing^≠^** | 0.8 | 1.9 | **97.2** |  | | **6.8** | | 4.3 | | 88.9 | |
| **Dancing^≠^** | 1.4 | 1.7 | **97** |  | | **7.6** | | 4.2 | | 88.2 | |
| **Sexual activity^≠^** | 4.5 | 1.4 | **94.1** |  | | **9.5** | | **10** | | 80.5 | |
| **Orgasm^≠^** | 1.1 | 0.8 | **98.1** |  | | **6.5** | | **10** | | 83.5 | |
| **Lack of sleep^≠^** | 56.6 | 1.1 | **42.3** |  | | **77.8** | | 0.8 | | 21.3 | |
| **Excess of sleep^≠^** | 8.4 | 1.1 | **90.5** |  | | **54.8** | | 0.4 | | 44.8 | |
| **Seizures at awakening^≠^** | 18.2 | 0.5 | **81.3** |  | | **40.6** | | 0 | | 59.4 | |
| **Seizures at falling sleep^≠^** | 11.4 | 0.3 | **88.3** |  | | **28.9** | | 5.4 | | 65.7 | |
| **Physical stress^≠^** | 29.5 | 0.9 | **69.6** |  | | **56.1** | | 0.8 | | 43.1 | |
| **Emotional stress^≠^** | 55.3 | 0.5 | **44.2** |  | | **81.6** | | 1.3 | | 17.2 | |
| **Fever^≠^** | 13.6 | 0.8 | **85.6** |  | | **30.1** | | 0.4 | | 69.5 | |
| **Hunger^≠^** | 12.5 | 1.1 | **86.4** |  | | **71.1** | | 0.8 | | 28 | |
| **Menstruation^≠^** | 18.9 | 0 | **81.1** |  | | **68.4** | | 0.5 | | 31.1 | |

*Statistical calculations were done among those who exposed to modulatory factors The* ***bold and underlined*** *ones show the statistically significantly high rates according to adjusted residuals (>2) and p<0.05 in Pearson chi-square’s. Decision ..to do.:Decision-making between different possible things to do. Decision..approaches: Decision-making between different approaches to a task. Provoc: Provocation. NoMod: No modulation. Inhib:Inhibition .^≠^:p≤0.001.* **^≠≠^***:p<0.05*

|  |
| --- |
